# Next Decade Research in Asthma: Broad Omics-Based Exploration Versus Targeted Airway Epithelium Studies

**DOI:** 10.3390/jcm14228186

**Published:** 2025-11-18

**Authors:** César Picado, Alberto Garcia de la Fuente, Ebymar Arismendi, Jordi Roca-Ferrer

**Affiliations:** 1 Institut d’Investigacions Biomèdiques August Pi i Sunyer (IDIBAPS), 08036 Barcelona, Spain; agarciadef@clinic.cat (A.G.d.l.F.); earismen@clinic.cat (E.A.); jrocaf@recerca.clinic.cat (J.R.-F.); 2 Faculty of Medicine, University of Barcelona, 08007 Barcelona, Spain; 3 Centro de Investigaciones Biomédicas en Red de Enfermedades Respiratorias (CIBERES), 28029 Madrid, Spain; 4 Department of Allergic Diseases, Hospital Clinic Barcelona, 08036 Barcelona, Spain; 5 Department of Respiratory Diseases, Hospital Clinic Barcelona, 08036 Barcelona, Spain

**Keywords:** asthma, airway, epithelium, cells, endotype, junction, omics, phenotype

## Abstract

Understanding asthma’s endotypes is key to advancing precision medicine. Using omics techniques on sputum, bronchial epithelium, and blood have revealed T2 and non-T2 asthma, each which have been further categorized into T2 and non-T2 subgroups. Despite advances in understanding asthma’s molecular complexity, many questions remain. Future research could either enhance current multiomics approaches with sophisticated bioinformatics or integrate hypothesis-driven research. It is now widely accepted that the airway epithelium starts and regulates the inflammatory cascade in asthma. If asthma originates in the altered epithelium, concentrating research on epithelial dysfunction is logical. This approach is likely more straightforward than analyzing the multitude of genes affected by the inflammatory cascade triggered by this disturbed airway epithelium. The airway epithelium comprises various cell types, including basal cells, club cells, ciliated cells, goblet cells, pulmonary neuroendocrine cells, tuft cells, and pulmonary ionocytes, which are connected by junctional complexes including tight junctions, adherens junctions, gap junctions, and desmosomes. The healthy airway epithelium helps support homeostasis, defend against threats, and regulate immunity through innate and adaptive systems. Chronic airway epithelial barrier dysfunction can instigate and propagate excessive immune responses. Knowing the cellular makeup and differentiation of the airway epithelium is vital for creating treatments to restore airway integrity in established asthma. A new consensus highlights focusing research on airway epithelial dysfunction as the main driver of inflammation, marking the start of the “epithelium era” in asthma research.

## 1. Introduction

Bronchial asthma is an airway condition marked by occasional symptoms of different intensities, such as shortness of breath, coughing, and excess mucus production, along with audible wheezing. These symptoms reflect a variable and reversible limitation of expiratory airflow linked to nonspecific airway hyperreactivity [1].

Chronic inflammation and increased mucus production cause airway narrowing in asthma. This inflammation involves various immune cells, such as eosinophils, neutrophils, lymphocytes, innate lymphoid cells (ILCs), and mast cells [1]. Asthma’s airway remodeling involves basal membrane thickening, subepithelial fibrosis, and smooth muscle hypertrophy [2]; however, it is not yet clear whether this remodeling precedes chronic inflammation, results from it, or both [2]. Inflammation and remodeling result in airway hyperreactivity, which can trigger asthma attacks induced by allergens, irritants, or physical exertion [1].

## 2. Asthma Heterogeneity: Phenotypes and Endotypes

Asthma shows various clinical characteristics, referred to as phenotypes, which are associated with distinct underlying causal mechanisms, known as endotypes [1].

### 2.1. Asthma Phenotypes

In 1947, FM Rackemann first classified asthma into two types: extrinsic (allergic) and intrinsic (nonallergic), with various subphenotypes to reflect its complexity [3].

Subsequent research proposed expanding phenotypes by considering factors such as age, disease duration, severity, frequency of exacerbations, respiratory function, biomarkers like blood immunoglobulin E (IgE) and eosinophilia, and comorbidities such as chronic rhinosinusitis with nasal polyps (CRSwNP), non-steroidal drug exacerbated respiratory disease (N-ERD), and obesity. These classifications incorporate data from underlying inflammatory processes, as determined through sputum or blood tests, in addition to clinical characteristics. These studies produced various phenotypic classifications that share some similarities but also exhibit differences in adults [4,5,6] and children [7,8].

The 2024 Global Initiative for Asthma (GINA) guidelines list common phenotypes, such as allergic asthma, nonallergic asthma, adult-onset asthma (often non-allergic), asthma with persistent airflow limitation, and obesity-associated asthma [9].

Attempts to better classify asthma’s clinical heterogeneity have not significantly advanced beyond Rackemann’s proposal [3]. He did not describe asthma in obese individuals or those with persistent airflow limitations because obesity was less common globally in the 1940s, and spirometry was not used.

### 2.2. Asthma Endotypes

To better understand asthma, numerous studies over the past two decades have focused on its pathobiological mechanisms using genetics, epigenetics, transcriptomics, proteomics, and metabolomics. These techniques have analyzed samples from patients’ airways (breath, sputum, bronchial brushing, bronchial biopsies, and bronchoalveolar lavage) and other compartments (blood and urine), considering different clinical characteristics and severity [10,11].

Endotype mechanisms have been identified through various clustering methodologies by performing statistical bioinformatic analyses on clinical, inflammatory (sputum and blood), and omics data [10,12].

Data from sputum and blood have been used to define phenotypes or endotypes, underscoring the ambiguity in the terminology employed to classify asthma pheno/endotype heterogeneity.

Asthma endotypes have been classified using various approaches, such as eosinophilic, non-eosinophilic, Th2-high, Th2-low, and non-Th2. The discovery that innate immune cells, like ILC2, can produce Th2-associated cytokines led to a shift from Th2 to T2 and non-T2 asthma. This manuscript will use the classifications referenced in the cited works.

Rather than serving as a systematic review, this article offers a focused evaluation of the most pertinent studies addressing asthma heterogeneity. The selection emphasizes research conducted by both national and international investigators, with particular attention to large-scale studies that have contributed to important insights.

PubMed papers describing asthma phenotypes and endotypes in adults using transcriptomic, proteomic, metabolomic, and metagenomic methods were selected. Papers focusing on the study of specific cells (T lymphocytes, macrophages, eosinophils, neutrophils, and mast cells) were excluded. Similarly, studies focused on a single phenotype (e.g., asthma in older adults) or endotype (e.g., severe asthma) were also excluded.

This review provides an overview of key omics studies classifying asthma heterogeneity, highlighting the complexity and challenges in translating findings to clinical practice.

#### 2.2.1. Endotyping Asthma Using Sputum

Researchers classify asthma endotypes by analyzing sputum cell ratios: eosinophilic (eosinophils ≥ 3%, neutrophils ≤ 61%), neutrophilic (neutrophils ≥ 61%), mixed (eosinophils ≥ 3%, neutrophils ≥ 61%), and paucigranulocytic (normal cell counts) [13]. However, inflammatory cell endotypes are unstable, with studies showing their patterns change over time [14,15,16].

Sputum eosinophil concentration helps guide asthma management and predict treatment outcomes [17]. Blood eosinophil counts moderately correlate with sputum eosinophilia and can serve as a practical substitute [17,18]. However, induced sputum analysis does not adequately reveal the cellular and inflammatory processes behind asthma. Therefore, more research was needed to understand respiratory tract inflammation mechanisms.

Many studies have examined asthma endotypes by comparing gene expressions in induced sputum. Transcriptomic analyses assess all RNA transcripts—including messenger, non-coding, and microRNA—in targeted cells or tissues.

Most transcriptomic studies in asthma rely on RT-qPCR and DNA microarrays, with RNA-Seq gaining attraction recently. Gene set enrichment and differentially expressed genes (DEGs) network analysis help reveal affected pathways. Gene Set Variation Analysis examines coordinated gene groups, offering broader insight into biological processes than single-gene approaches. Whole-genome studies assess hundreds to thousands of DEGs to uncover asthma mechanisms, while analytical tools compare DEGs between controls and patients [19].

RT-qPCR measured IL-4, IL-5, and IL-13 expressions in sputum cells from 36 asthmatic patients and eight controls. Asthmatics showed significantly higher cytokine levels, with strong positive correlations among IL-4, IL-5, and IL-13. Control data defined a threshold to classify T2-high versus T2-low asthma; 67% of asthmatics were T2-high, associated with increased eosinophils and more severe, treatment-resistant asthma needing biologics [20].

A panel of T-cell and innate cytokines was measured in induced sputum using RT-qPCR, including Th1, Th2, Th17, and innate markers. Patients were classified as “cytokine-low” or “cytokine-high” if mRNA levels fell outside the control group’s 10th–90th percentile. Five clusters emerged: (1) high IL-5, IL-10, IL-25, IL-17A, IL-17F; (2) high IL-5/IL-10, normal IL-17F; (3) high IL-6; (4) high IL-22, with half also high IL-1β; (5) normal cytokine levels [21]. Clusters 1 and 5 showed higher sputum eosinophil percentages; Clusters 1 and 4 had more neutrophils, while Cluster 5 had fewer. The “IL-4- or IL-13-high” profile appeared evenly across all clusters, making type 2 versus non-type 2 classification unclear [21].

Whole-genome differential expression analysis of induced sputum in patients with asthma identified a six-gene signature. High expression of the Charcot-Leyden crystal protein (*CLC)*, carboxypeptidase A3 (*CPA3)*, and deoxyribonuclease I-like 3 (*DNASE1L3)* genes defined eosinophilic asthma, while high expression of *IL1β*, alkaline phosphatase, tissue-nonspecific isozyme (*ALPL)*, and chemokine (C-X-C motif) receptor 2 (*CXCR2*) genes characterized non-eosinophilic asthma and predicted inhaled corticosteroids (ICS) response [22].

The same research group found elevated gene expression of NLRP3 (nucleotide-binding domain and leucine-rich repeats containing pyrin domain 3), caspase-1, caspase-4, caspase-5, and IL-1β in sputum macrophages from patients with neutrophilic asthma. IL-1β protein levels correlated with sputum IL-8 levels. These findings suggest that the NLRP3 inflammasome regulates neutrophilic inflammation in asthma through IL-1β production [23].

A study using whole-genome gene expression profiling identified three transcriptional asthma phenotypes (TAPs) linked to clinical status and airway inflammation. TAP1 was enriched with eosinophils, showed high fractional exhaled nitric oxide (FeNO), severe airflow obstruction, and poor asthma control. TAP2 was neutrophil-enriched and had severe airflow obstruction. TAP3 featured normal sputum eosinophil and neutrophil levels, more macrophages, and near-normal lung function, resembling paucigranulocytic asthma. Most genes upregulated in TAP1 and TAP2 related to immune and inflammatory responses, while IL-1 and TNF-α/NF-kB pathways were specifically elevated in TAP2 and associated with neutrophilic inflammation [24].

A related study analyzed sputum from participants with varying asthma severity and healthy controls, using Western blotting to measure p38 activation. Researchers identified four gene networks among 123 genes that were differentially expressed in severe asthma, with thirty genes differing significantly compared to controlled asthma and healthy individuals. Genes linked to the p38 signaling pathway were more abundant in severe asthma, and higher p38 phosphorylation was associated with neutrophilic airway inflammation [25].

A study classified T2 asthma cases into T2-low, T2-high, and T2-ultrahigh subgroups. Those with T2-ultrahigh asthma were older and showed elevated expression of a gene signature linked to CD11b1/CD1032/IRF41 dendritic cells. Cluster analysis revealed three subnetworks within the T2 network; Subnetwork 1 encompassed all T2 cytokine genes (IL-4, IL-5, IL-13), three mast cell/basophil-specific genes (*CPA3*, HDC, *MS4A2*), and the transcription factor GATA_2 involved in basophil and mast cell development [26].

Subnetwork 2 contained *CEBPE* and *GATA1*, both essential for eosinophil development, and featured *IL1RL1* as a hub gene involved in IL-33 signaling and T2 inflammation regulation. In contrast, Subnetwork 3 included genes characteristic of inflammatory dendritic cells (IDECs): *CD1A*, *CD1B*, *CD1C*, *CD1E*, *RAMP1*, *CD207*, *F13A1*, *CCL17*, and *FCER1A* [26].

Further analysis revealed a strong correlation between the T2 network and *CD11B* (a dendritic cell marker), *ZBTB46* (zinc finger and BTB domain-containing 46), and *FLT3* (fms-related tyrosine kinase 3), genes associated with classical dendritic cells (cDCs). Additionally, analysis of the genes in this cDC network showed evidence of activation via NF-KB and increased IL-2 production, particularly in patients with T2-ultrahigh asthma [19]. Collectively, these data support that the T2 cytokine signal in the airways of patients with T2-high asthma results from the complex interaction of multiple innate immune cells, including eosinophils, mast cells/basophils, and cDCs [26].

T2-low asthma features reduced CD8+ cytotoxic T cells, which correlate negatively with IL-6 and obesity-related inflammation. This may contribute to poor corticosteroid response in patients with sub-clinical viral infections [26].

A U-BIOPRED study used transcriptomic analysis of asthma sputum and found three transcriptome-associated clusters (TACs). Individuals in TAC1 had high sputum eosinophils and severe asthma with frequent exacerbations. This cluster expressed *IL33R*, *CCR3*, and *TSLPR* genes, and showed the strongest gene signatures for IL-13-induced epithelial cells and ILC2 transcripts, both linked to the T2 signature [27].

TAC2 was marked by high sputum neutrophilia, increased *TNF-α* expression, and inflammasome activation in patients with moderate airway obstruction and eczema. Though neutrophilia and inflammasome activity were key features, elevated *IL-13T2* also suggested both T1 and T2 involvement [27].

TAC3 is linked to paucigranulocytic asthma and increased expression of metabolic and mitochondrial pathway genes, especially those for oxidative phosphorylation. Clinically, patients typically have mild to moderate asthma, occasional exacerbations, and moderate airflow limitation [27].

Researchers tracked U-BIOPRED severe asthma patients for a year and found that 55% maintained stable endotypes (TACs), while 45% changed, mainly from TAC1 or TAC3 to TAC2. In those moving from TAC1, sputum eosinophil counts dropped from 25.2% to 16.3%. These shifts suggest that corticosteroid use, impacting eosinophilic inflammation, may drive changes in TAC status [28].

Transcriptomic endotypes of asthma (TEA) were analyzed using gene expression in induced sputum, revealing three clusters. Clusters 1 and 2 contained most severe cases, marked by frequent hospitalizations, intubations, and poor lung function; Cluster 3 comprised patients with milder disease and normal lung function. DEGs distinguished each TEA cluster from controls: clusters 1 and 2 showed downregulation of genes like EXOSC9 and SNAPPC5 (involved in RNA processing), and upregulation of NRCAM, PCLO, and SLC4A4 (neuron function). In Cluster 3, DEGs such as DNAH17 and DEFB1 were upregulated. The clusters were distinct, though some overlap existed, particularly between Clusters 1 and 2 [29].

A U-BIOPRED cohort study identified 10 sputum proteome clusters: three highly eosinophilic, three highly neutrophilic, two highly atopic, and two smaller mild endotypes. Ten proteins, including histone H4 and vitronectin, predicted eosinophilic clusters. Highly neutrophilic clusters featured histone H4, azurocidin, and coronin-1A, while high uteroglobin and clustering levels marked the highly atopic group [30].

Recent analyses of sputum samples from the U-BIOPRED and ADEPT cohorts found that Pregnancy-associated plasma protein A (PAPP-A) predicts eosinophilic asthma (≥1.5% cutoff), and cathepsin G is associated with eosinophilic asthma at ≥3%. No proteins correlated with neutrophilic asthma at ≥73.6%, but carbonic anhydrase 1, CD23, and CD27 were linked at ≥60%. The functions of these proteins in airway diseases need further study; cathepsin G may influence inflammation through chemokine, cytokine, and receptor modulation [31].

#### 2.2.2. Endotyping Asthma Using Bronchial Biopsies and Airway Brushing

Woodruff and colleagues used whole-genome microarray expression profiling on bronchial airway brushings from adults with asthma. In their study, the researchers found that asthma patients showed distinct activation of the epithelial genes periostin (*POSTN*), chloride channel regulator 1 (*CLCA1*), and serpin peptidase inhibitor clade B member 2 (*SERPINB2*) when compared to healthy controls [32]. Furthermore, they showed that IL-13 directly regulates these genes in vitro [32].

Two subgroups of asthmatics, Th2-high and Th2-low, were identified through unsupervised hierarchical clustering of the expression of three Th2 cytokine-induced genes. The addition of either dexamethasone or budesonide to the cultured epithelial cells inhibited the effects of IL-13. Additionally, the induction of MUC5AC and MUC2 and the repression of MUC5B were seen, which are specific to Th2-high asthma [33].

A recent study analyzed 808 bronchial epithelial cell (BEC) samples from 12 cohorts across six countries. Discovery was conducted using seven datasets, while validation relied on four separate datasets. The analysis revealed 505 DEGs in asthma BECs compared with healthy controls, with most genes showing consistent expression across validation datasets. Pathway analysis identified 21 significant pathways, including those related to cell proliferation and sex hormone response, supporting links between sex differences, obesity, and asthma. After co-normalization, 359 genes were retained, resulting in four distinct clusters [34].

Cluster 1 was the largest and primarily included healthy subjects. Clusters 2, 3, and 4 contained most patients with asthma. Cluster 2 mainly consisted of individuals with mild to moderate asthma, while Clusters 3 and 4 primarily included individuals with severe asthma [34].

Genes related to T2 inflammation (*POSTN*, *SERPINB2*, *CLCA1*, *CCL26*, *ITLN1*, *MS4A2*, *CPA3*, *ALOX15*, *IL18R1*, and *GATA2*) showed higher expression in Clusters 2 and 4 than in Clusters 1 and 3. Cluster 4 exhibited higher gene expressions than Cluster 2, while Clusters 1 and 3 had levels comparable to those of the controls [34].

Gene expressions of *FKBP5*, *HSD11B2*, and *CST1* varied in response to steroid exposure. Healthy subjects had low expression, whereas patients with asthma, particularly those in Clusters 1 and 3, showed high levels. *FKBP5* expression rose with glucocorticoids, being still low in healthy and steroid-naïve Cluster 4 patients but was high in Cluster 3 patients who used high doses of inhaled corticosteroids [34].

Patients in Cluster 1 had less severe asthma than those in Clusters 2, 3, and 4, with Cluster 4 being the most severe. Clusters 1 and 2 had higher FEV_1_ (Forced Expiratory Volume in 1 Second), fewer exacerbations, less bronchodilator reversibility, lower FeNO, IgE, and eosinophils than Clusters 3 and 4. Clusters 3 and 4 mostly included adults with severe asthma; Cluster 4 had more exacerbations, higher bronchodilator reversibility, FeNO, and eosinophils than Cluster 3. Despite both Clusters 2 and 4 showing high T2 inflammation gene expression (*POSTN*, *SERPINB2*, and *CLCA1*), their clinical severity markers differed significantly. Clusters 2 and 4 also had higher FeNO than Clusters 1 and 3 [34].

The *SCGB3A1*, *C3*, and *ZMAT2* genes exhibited the lowest expression in Clusters 2 and 4, higher in Cluster 3, and the highest in Cluster 1. Cluster 3 had the most blood and BAL neutrophils, while Cluster 1 had the most sputum neutrophils. These findings suggest that patients in Clusters 1 and 3 express T2-low asthma-related genes. The gene expression differences between these clusters indicate distinct clinical characteristics, aiding in reducing patient heterogeneity in T2-low asthma [34].

Pathways related to sex hormones (androgens and estrogens) were significantly upregulated in patients with asthma. Among the 359 genes examined, 43 showed significant differences in expression between male and female asthma patients. Out of the 43 genes, 10 showed significant differences in expression in at least one cluster. Importantly, eight out of these ten genes, including the T2-low asthma genes *ZMAT2* and C3, showed differential expression within Cluster 3. These findings align with established associations between severe asthma, females, and obesity [34].

In another recent study, the transcriptome derived from bronchial biopsies and epithelial brushings of 107 subjects with moderate to severe asthma was annotated through gene set variation analysis using 42 gene sets encompassing 2431 gene signatures. Specifically, these included six gene sets for immune cell type-specific gene expression, six for evolutionarily conserved transcriptional signatures for Th1 and CD8 memory T-cell differentiation, and five for effector CD4+ T-cell differentiation, including Th2, Th17, and regulatory T-cell subsets. Additionally, six gene sets addressed chronic effects of oxidative stress in response to ozone in a mouse model and genes associated with chronic obstructive pulmonary disease (COPD) in human lung samples. Seven gene sets were derived from peripheral blood mononuclear cells in four other autoimmune diseases, seven for asthma-specific mechanisms driven by T cells, and five from human lung biopsy, airway smooth muscle cells, and peripheral blood mononuclear cells in response to corticosteroid treatment [35].

No significant differences or marginally significant expressions were observed between individual genes or gene sets from epithelial brushings and bronchial biopsies when comparing severe and non-severe asthma cases. In both sample types, CD44 and NELFE, as components of the corticosteroid insensitivity signature, were associated with a subset of Th2 signature genes, including *CCL26*, *IL1R2*, *CST2*, and *ATP5J* [35].

Unsupervised clustering of gene sets applied to biopsies identified a cluster (referred to as Cluster A) characterized by higher submucosal eosinophil counts, elevated levels of nitric oxide in exhaled breath, and increased oral corticosteroid use. A comparison of Cluster A with non-A showed that 26 out of 42 gene sets had differences in expression [35].

Further analysis proved that nine gene sets expressed in bronchial and brushing samples enabled differentiation into two distinct asthma subtypes associated with high expression of T-helper cell type 2 cytokines (Groups 1 and 3). Group 1 displayed the highest numbers of eosinophils in the submucosa, higher FeNO, and greater oral corticosteroid use. Increased levels of eosinophils were observed in both the blood and sputum. This group showed the nine signatures in both bronchial and epithelial brushing cells. Group 3 participants showed elevated levels of sputum eosinophils and a high body mass index (BMI), expressing the nine signatures solely in epithelial brushing samples. Conversely, most participants in Groups 2 and 4 were non-eosinophilic. Group 4 showed significantly lower enrichment of signatures representative of various immune pathways, such as Th2, Th1, Th17, and neutrophils in sputum compared with Group 1, indicating a dominant paucigranulocytic asthma phenotype in Group 4 [35].

#### 2.2.3. Endotyping Asthma Using Blood

Proteomic profiles were examined in serum samples (*n* = 574) collected from two patient cohorts, U-BIOPRED and ADEPT, which included individuals with mild to moderate as well as severe asthma. The study aimed to identify sparse sets of proteins associated with either eosinophilic or neutrophilic asthma, both with and without adjustment for established clinical factors. Thirteen serum proteins were found as being associated with eosinophilic asthma, including PAPP-A, TARC/CCL17, ALT/GPT, IgE, CCL28, CO8A1, and IL5-Rα. When adjusted for clinical factors, these yielded an area under the curve (AUC) of 0.84 (95% CI: 0.83–0.84), compared with 0.62 (95% CI: 0.61–0.63) for clinical factors alone. Upon lowering the eosinophil serum classification threshold from 300 to 150/μL, PAPP-A and IgE remained consistently selected, whereas IL5Rα and TARC/CCL17 were no longer prominent. Twelve serum proteins were found to be associated with neutrophilic asthma, of which five (MMP-9, EDAR, GIIE/PLA2G2E, IL-1-R4/IL1RL1, and Elafin) enhanced clinical models, increasing the AUC from 0.63 (95% CI: 0.58–0.67) to 0.89 (95% CI: 0.89–0.90. The thirteen serum proteins associated with eosinophilic asthma showed pathways related to cell growth, differentiation, proliferation, and T helper 2 (Th2) immune responses, potentially involving integrin-mediated and extracellular matrix (ECM) signaling. Conversely, serum proteins correlated with neutrophilic asthma were linked to airway remodeling pathways (MMP-9), neutrophil recruitment, and included one possible anti-inflammatory protein, Elafin [32].

Transcriptional profiles from peripheral blood mononuclear cells identified four clusters. Cluster 1 (smoking asthma with neutrophilic inflammation) showed lower FEV1% and more smoking history with higher sputum neutrophils. Cluster 2 (severe eosinophilic asthma) had more eosinophils in sputum and blood, plus severe airflow limitation. Cluster 3 (early onset atopic asthma with normal lung function) comprised younger subjects with atopic features. Cluster 4 (late onset mild asthma) included mostly women with almost normal lung function. Genes upregulated in Cluster 1 (*HLA-DRA, HLA-DRB3, CFD, SERPINB12*, *PPBP*, and *GNG11*) are engaged in the Th17, Th1, and Th2 pathways and cell activation. Clusters 2 and 3 presented few differences, and Clusters 1, 2, and 3 had no major differences from Cluster 4 [36].

A recent study examined baseline and clinical characteristics, as well as inflammation-related plasma proteins, in relation to eosinophil and neutrophil blood concentrations among subjects with and without asthma. The expression levels of 92 protein biomarkers were quantified using the Proseek Multiplex Inflammation Panel (Olink Biosciences). Among the subjects with asthma, 21.8% were identified as having eosinophilic asthma and 20.5% as having neutrophilic asthma. Eosinophilic asthma exhibited a distinct clinical phenotype characterized by higher proportions of eczema and allergen sensitization, which was not seen in neutrophilic asthma [37].

Most plasma proteins associated with high eosinophil and/or neutrophil blood concentrations in subjects with asthma demonstrated similar associations in those without asthma. Individuals with eosinophilic asthma had notably higher MMP10 levels compared to control subjects who also had high eosinophil counts. Metaloproteinasa (MMP) 10, a metalloproteinase from the extracellular matrix (ECM), has been previously found overexpressed in bronchial biopsies from asthmatic subjects with eosinophilic airway inflammation, underscoring its role in airway remodeling. CCL4, also known as macrophage inflammatory protein-1β (MIP-1β), is produced by macrophages, dendritic cells, lymphocytes, and epithelial cells in response to infection and has been linked to acute neutrophilic inflammation. Earlier research reported increased concentrations of CCL4 in bronchoalveolar lavage fluid (BALF) and an association with disease severity in adult asthmatics [37].

## 3. Multiomics

Multiomic analysis is being used in asthma research to study the disease’s complex nature. By combining data from various omic platforms, it aims to create more comprehensive models that illustrate the intermolecular interactions in different pathways of asthma pathogenesis [38].

In a study conducted by the U-BIOPRED consortium, adults with severe asthma were categorized into two clusters (C1 and C2) based on their sputum microbiome. The C2 cluster showed sputum neutrophilia and microbial imbalance, characterized by an increased presence of pathogenic bacteria, particularly *Haemophilus influenzae* and *Moraxella catarrhalis*, in comparison with the C1 cluster [39].

A subsequent multiomic study compared the C1 and C2 clusters with patients having mild to moderate asthma and healthy controls, using sputum omics, including eicosanoids, transcriptomics, proteomics, and metagenomics. C2 subjects had significantly higher levels of sputum 11-dehydrothromboxane B2, prostaglandin E2 (*PGE*_2_), and cyclooxygenase 2 (*COX-2*) gene expression than C1 subjects. Moreover, levels of 15-hydroxyeicosatetraenoic acid (HETE), leukotriene E4 (LTE_4_), and prostaglandin D2 were higher in C2 than in individuals with mild to moderate asthma or healthy controls [40].

Paired microarray gene expression data revealed that C2, with respect to C1, displayed upregulated pathways related to immune regulation and inflammation. For example, TNF-α and related regulatory genes, ILs and associated regulatory genes, Toll-like receptors (e.g., TLR2, TLR4, and TLR10), and inflammatory genes (e.g., *LRP3*, *NLRP12*, and *NLRC5*). Conversely, the C2 cluster showed downregulated pathways for cell growth, proliferation, DNA repair, oxidative phosphorylation (OXPHOS), tricarboxylic acid cycle, and pathways, suggesting impaired cellular repair functions and mitochondrial dysfunction [40]. Paired sputum proteomic data found 36 differentially abundant proteins between C2 and C1. Upregulated proteins in C2 were related to neutrophilia, inflammation, and/or Th17- and Th1-mediated pathways [40].

A recent U-BIOPRED study analyzed six-omic data types from sputum samples of 57 patients with severe asthma, 15 with mild-moderate asthma, and 13 healthy volunteers. Five stable omics-associated clusters (OACs) were identified. OAC1 and OAC5 included mild-moderate asthmatics with good lung function and paucigranulocytic inflammation; OAC5 also had high atopy and allergic rhinitis (AR) rates. OAC3 consisted solely of severe asthmatics with high sputum eosinophilia. OAC2 and OAC4 showed high sputum neutrophilia and mostly severe asthma; OAC4 had more ex/current smokers. OAC2 had microbial dysbiosis with abundant *Moraxella catarrhalis* and *Haemophilus influenzae*, while OAC4 was linked to IL-22 cytokine activation pathways [41].

## 4. Are Multiomics the Future Asthma Research?

Studies employing omics methodologies have provided insights into the underlying molecular mechanisms of asthma endotypes. By integrating substantial amounts of data, these studies aim to minimize potential biases that are theoretically more frequent in clinically driven approaches. Omics-based studies enable a deeper exploration of the molecular mechanisms regulating different cellular inflammatory types. Asthma endotypes are grouped as Th2-high and Th2-low, which eventually resulted in the classification of T2 and non-T2 (or low T2) categories. However, no endotype or subendotype relates to a single pathway, and there is significant overlap between T2 and non-T2 subtypes. In addition, inflammatory endotypes are dynamic and shift during exacerbations, therapy adjustments, and other factors such as seasonal changes and weight gain [42,43,44,45].

Studies using omics have certain limitations, including: (1) Many studies employ a cross-sectional design, which does not allow for the assessment of the temporal stability of endotypes. Only a few cluster studies include proper validation. (2) Replication and comparison of endotypes across different studies may be limited by the choice of variables used for clustering. Different approaches result in varying phenotypes with limited overlap and distinct clinical characteristics. (3) Diverse cohorts hinder the identification of subendotypes. (4) Small clusters may lead to overfitting and a lack of generalization. (5) Geographic location, ethnicity, and environmental exposures affect asthma manifestation, limiting the generalizability of the findings. (6) The cellular makeup of bulk samples can confound results; thus, some studies employ cell deconvolution methods. (7) Omics data help in patient stratification; however, their application to personalized treatment is still uncertain [10,11,12,46].

Optimistic perspectives suggest that advancements in understanding the mechanisms of asthma, primarily achieved through omics methods, are now ready for application in the personalized management of the disease in real-world settings [47]. However, a more critical analysis reveals various challenges: (1) The results obtained from omic studies have not yet been included in recent asthma reviews and updates [1]. (2) The latest asthma treatment guidelines do not incorporate omics studies. They rely on evaluating IgE-dependent symptoms, FeNO levels, and blood eosinophil counts [9]. (3) Omics studies aim to enable precision medicine; nonetheless, this goal stays unmet in clinical practice. Electing the most appropriate biologic remains challenging due to overlapping phenotypic features and the limited number of validated biomarkers. A recent study highlighted the variability in asthma treatment choices among physicians for the same patient, highlighting the lack of precision in current management strategies [48].

With the data from the last two decades, it is crucial to review the research model focused on omics to explain asthma’s heterogeneous clinical expression and its varied inflammatory and non-inflammatory patterns.

Eight years ago, Belgrave et al. [49] cautioned against using only “big data” to formulate hypotheses, emphasizing that such data should not be considered self-explanatory. They recommended a balanced approach that combines data-driven and hypothesis-driven methods. Their proposal did not gain significant attention from the scientific community, which opted to prioritize complex omics techniques and advanced bioinformatic methods over hypothesis-driven research.

Considering the current results, should we integrate omics with hypothesis-driven research, or will collecting more multiomics data improve our understanding of asthma’s complexity?

If the initial option is selected, which hypothesis might be used to inform the identification of asthma endotypes using a hypothesis-driven research approach?

## 5. The Future of Asthma Research: Targeted Studies of Airways Epithelium

It is well proved that the airway epithelium plays a significant role in asthma development. Common structural changes such as fragile epithelial cells, thickening beneath the membrane, and muscle cell hypertrophy are observed in children even before asthma symptoms appear or lung function decreases. This suggests that early alterations in the epithelium play a role in the development of asthma [2,50,51].

The airway epithelium, like the skin and gut epithelia, protects against environmental agents such as infections, toxins, irritants, extreme temperatures, and allergens. Beyond serving as a physical barrier, it uses cilia and mucus for defense and engages both innate and adapted immunity. Mucus traps particles that cilia propel out of the airways, and it secretes lysozyme, lactoferrin, and peptides like β-defensins and cathelicidins with antimicrobial capacity [2,50,51].

Airborne stimuli trigger the damaged mucosal epithelium to release various molecules that affect the activity of resident immune and environmental cells. Signals from the mucosal barrier are essential factors that can alter the function of innate and adaptive effector cells [2,50,51].

Studying the role of genes with altered expression in asthma involves extensive research. It may be practical to focus on the genes that trigger the complex inflammatory response, based on the idea that most gene activations result from the dysregulation of a smaller group of genes. For instance, examining the role of thymic stromal lymphopoietin (TSLP), an epithelial cytokine that has multifaceted effects on the initiation and persistence of asthma inflammation and pathophysiology, is more practical than studying the numerous genes whose expression changes following TSLP activation. Tezepelumab—a monoclonal antibody that blocks stromal lymphopoietin—may modulate airway inflammation more extensively than other biologics that only block specific downstream components of the inflammatory cascade [52].

Future asthma research should focus on the epithelium, with the next challenge being the identification of specific biomarkers for each airway epithelium endotype to better define asthma heterogeneity [53,54].

Single-cell RNA sequencing (ScRNA-seq) and lineage tracing have revealed that the airway epithelium includes common cell types, such as basal cells (BCs), club cells (CC), ciliated cells CLCs), and goblet cells (GC), as well as rare types, such as pulmonary neuroendocrine cells (PNEC), tuft cells (TFc), and pulmonary ionocytes (PI) [55,56,57].

### 5.1. Basal Cells in Health and in Asthma

BCs attached to the basement membrane are essential for epithelial regeneration, serving as stem cells that can self-renew and differentiate into GCs, CLCs, CCs, and TFcs, as well as PIs [58,59]. Following injury to the airway epithelium, BCs divide and give rise to suprabasal cells, a common stage in CLC and GC differentiation, or TFcs. Research suggests at least two transcriptionally distinct subpopulations of BCs in human airways: one functioning as stem cell progenitors (self-renewal) and the other committed to luminal cell differentiation [60,61]. They can be identified by markers such as cytokeratin 5 and 14 (KRT5/14), and transformation-related protein 63 (Trp-63), respectively [60,61].

The NOTCH pathway plays a role in BC differentiation, with its activation leading to the differentiation of BCs into secretory cells, whereas inhibition of NOTCH1/2 signaling results in differentiation into multiciliate cells [62].

BCs also contribute to immunoregulation by producing cytokines and chemokines in response to inflammation, aiding local immune responses [51,63]. Upon epithelial barrier disruption, BCs secrete pro-inflammatory factors, such as IL-1β, IL-6, IL-11, and TNF, which activate dendritic and T cells, and recruit macrophages, neutrophils, and T lymphocytes, thus playing a role in airway diseases [64]. Immune dysfunction may cause cytokine storms, worsening inflammation and tissue damage [64].

In asthma, the airway epithelium is less differentiated, with more cytokeratin-5 positive BCs and increased p38 MAPK phosphorylation [65]. The number of BCs directly correlates with epithelial thickness [66].

### 5.2. Club Cells in Health and in Asthma

CCs, previously known as Clara cells, can also function as stem cells, aiding in epithelial repair by differentiating into CLCs or GCs, and differentiating into BCs if needed [67,68,69]. They also function as secretory cells, primarily expressing Scgb1a, which encodes uteroglobin—an abundant protein in airway lining fluid [67,70]. Uteroglobin, also called Clara cell protein 16 (CC16), exhibits anti-inflammatory and immunosuppressive properties by inhibiting neutrophil and monocyte activity, as well as interferon gamma (IFN-γ), tumor necrosis factor alfa (TNF-α), and IL-1 production [67,71].

Studies have examined the role of CC16 produced by CCs in patients with asthma and mouse models.

Low serum CC16 has been associated with increased asthma morbidity in both childhood and adulthood. This association is seen in cases of both atopic and nonatopic asthma and is particularly pronounced in patients with frequent asthma symptoms and reduced lung function. Furthermore, children with the lowest levels of CC16 are more likely to experience persistent symptoms into adulthood [72].

Reduced CC16 mRNA expression in BECs, associated with increased T2 pathway genes, has been noted [73]. Lower CC16 mRNA levels are associated with decreased lung function, higher corticosteroid use, and more frequent asthma exacerbations due to increased T2 inflammation [73]. Additionally, low circulatory CC16 levels correlate with enhanced eosinophilic inflammation, greater airway hyperresponsiveness, and faster decline in FEV1 over time [74,75].

Being overweight or obese was inversely associated with CC16 levels in both asthmatic and non-asthmatic populations. The percentage of CC16-expressing cells was reduced in the small airways of both mice and humans with obesity. Analysis revealed significant contributions of circulatory CC16 in the association between body mass index (BMI) and asthma severity, as well as the required dose of inhaled corticosteroids. These findings suggest that low CC16 production by CCs may contribute to the association of obesity with asthma that responds poorly to inhaled corticosteroids [76].

CC16 effectively mitigates damage to AECs caused by PM2.5 (particulate matter with a diameter of less than 2.5 μm suspended in the air) in asthmatic mice. CC16 suppresses pyroptosis and inflammation [77]. Furthermore, CC16 significantly reduces Th2-type allergic inflammation in asthma by modulating lung dendritic cell subsets, specifically CD11b + CD103-DCs, in the lungs of asthmatic mice [78].

High mobility group box 1 (HMGB1) is an inflammatory mediator released from injured and dying cells. In a mouse model of allergic asthma using the house dust mite, increased HMGB1 expression was seen in the airways. CC16 reduces HDM-induced airway inflammation and damage by inhibiting epithelial cell apoptosis in an HMGB1-dependent manner, proving its anti-asthmatic effects [79].

These findings collectively support the potential therapeutic role of CC16 in alleviating airway inflammation.

A recent study found that dupilumab, a human IgG4 monoclonal antibody that inhibits IL-4 and IL-13 signaling by binding to the shared IL-4 receptor subunit alpha (IL-4Rα) of IL-4 and IL-13 receptor complexes, significantly reduced mucus plugs and airway wall area, while increasing serum CC16 levels. Changes in CC16 correlated with improvements in exhaled nitric oxide, quality-of-life score, forced expiratory volume 1 s (FEV_1_), mid-expiratory flow, and mucus plug score. These findings suggest that restoring normal CC16 function contributes to the beneficial effects of dupilumab in treating severe asthma [80].

### 5.3. Ciliated Cells in Health and in Asthma

CLCs help maintain airway homeostasis by removing microorganisms, debris, and mucus via mucociliary clearance (MCC) [81]. Originating from CCs or GCs, their differentiation is governed by transcription factors such as NOTCH, MYB, GMNC, and Foxj1 [82,83,84,85]. CLCs are found throughout the airways, with at least two distinct subsets along the proximal–distal axis [81]. Mutations in over 29 cilia-related genes can cause primary ciliary dyskinesias (PCD), a group of chronic lung disorders [86].

Ciliated epithelium shedding in asthma patients has been observed in autopsy [87], airway biopsies [2], sputum [65], and bronchoalveolar lavage [88].

Airway biopsies from children with asthma in remission and two fatal cases showed mucus plugs containing degenerate epithelial cells, macrophages, and cell fragments. CLCs displayed cytoplasmic blebs and abnormal cilia [87].

Moderate and severe asthma is linked to increased dyskinetic cilia, lower ciliary beat frequency, and impaired MCC [88,89]. Asthma patient sputum inhibits cilia beating in bronchial epithelial explants, whereas sputum from controls and most non-asthmatics does not [90].

The Th2-type cytokines IL-4 and IL-13 play key roles in asthma-related epithelial changes [91]. IL-4 drives inflammation by promoting Th2 cell differentiation, increasing IgE production, and attracting eosinophils [91]. IL-13 mainly causes airway remodeling, leading to GC hyperplasia, excessive mucus production, and smooth muscle hypertrophy [92]. It also impairs ciliary transport by binding mucus gels to the epithelium via MUC5AC [93]. Both cytokines contribute to fibrosis by influencing lung fibroblasts and ECM dynamics [91].

Applying IL-4 and IL-13 to airway epithelium cultures reduces CLC numbers, cilia per cell, and ciliary beat frequency [94]. IL-13 disrupts ezrin expression and its apical localization, decreasing basal bodies [95]. These changes are linked to reduced FoxJ1 expression via lower promoter activity and less ezrin [95].

Adding dupilumab to IL-13-treated cell cultures partially restored cilia in asthma patients, indicating that enhancing CLC function may help explain the therapy’s benefits [96].

### 5.4. Goblet Cells in Health and in Asthma

GCs, named for their shape, are the primary mucus-producing cells in the airways, working with CLCs to ensure effective MCC [97]. Mucus consists of electrolytes, metabolites, fluids, antimicrobial products, and mucins, such as MUC5AC and MUC5B [97,98,99]. Like CLCs, GCs originate from CCs via alternative transcriptional pathways regulated by SAM pointed domain-containing Ets-like factor (SPDEF) and forkhead boxA3 (Foxa3) [97,98].

The GC lineage is now divided into two groups: goblet-1 cells, which produce mucus, and goblet-2 cells, which secrete proteins (orthologues of zymogen granule protein 16) that bind and aggregate Gram-positive bacteria [55,98,100].

Patients with asthma have more GCs in their airways than healthy subjects, leading to mucus hypersecretion [101]. AECs respond to pollutants and damage by producing epidermal growth factor receptor ligands, which trigger mucus secretion [102]. Mucus hypersecretion is linked to chronic rhinosinusitis, nasal polyps, airway obstruction, poor asthma control, and exacerbations [103,104].

IL-13 and IL-4 are involved in mucus overproduction in asthma. IL-13 leads to GC hyperplasia and increased MUC5AC expression during epithelial differentiation, which results in a higher number of mucus-producing cells [105,106]. A characteristic of asthma is the altered MUC5B:MUC5AC ratio, with MUC5AC being more predominant in asthmatic sputum [107,108]. IL-13 can interfere with MCC by binding mucus gels to the epithelium through MUC5AC [92]. Additionally, IL-4 contributes to mucus hypersecretion by upregulating the mucin genes *MUC5* and *MUC4*, with *MUC4* being regulated via Janus kinase 3 (JAK-3) signaling [109,110,111].

In contrast to the demonstrated effect of dupilumab on cilia in cell culture having IL-13, the biologic did not cause any significant effect on MUC5AC expression [96].

A recent study has shown that dupilumab significantly reduces mucus hypersecretion in patients with moderate-to-severe uncontrolled asthma [112]. This likely involves multiple mechanisms, including improved IL-4/IL-13-dependent MCC.

### 5.5. Pulmonary Neuroendocrine Cells in Health and in Asthma

PNECs are rare epithelial-resident cells that connect the immune and nervous systems by sensing airway activity and secreting neuropeptides to stimulate immune responses [113]. Discovered in the 1950s, these cells comprise approximately 0.5% of the epithelial cells in human airways [114]. PNECs can be solitary or form neuroepithelial bodies [80]. They may also act as stem cells during tissue repair [113,115] and potentially have chemosensory functions due to their expression of olfactory receptors [116].

PNECs affect immunity by releasing chemicals, such as serotonin, calcitonin gene-related peptide (CGRP), and bombesin-related peptides [113]. These substances interact with structural and immune cells. Bombesin recruits mast cells to the airways and can induce bronchoconstriction [113,117,118]. Serotonin can also cause bronchoconstriction, and CGRP can induce mucus secretion in the airways from both glands and GCs [113,119,120,121,122].

Mice without PNECs (Ascl1CKO mice) had fewer ILC2s, eosinophils, and Th2 cells compared with control mice after ovalbumin (OVA) challenge. These changes were associated with decreased IL-5 and IL-13 expressions in PNECs located near ILC2s at airway branch points. Additionally, PNECs stimulate ILC2s through CGRP and induce GC hyperplasia via neurotransmitter gamma-aminobutyric acid (GABA). Introducing a mixture of CGRP and GABA into Ascl1-mutant airways restored both immune and glucocorticoid responses [123].

Furthermore, samples from patients with asthma showed an increase in PNEC number in the proximal bronchiole or distal respiratory bronchiole compared with control samples [124].

Another study involving OVA-induced asthmatic mice also found hyperplasia of CGRP-immunoreactive PNECs along with an increased total lung CGRP content. Upon antigen challenge, the concentration of plasma CGRP was transiently upregulated, while CGRP immunoreactivity within PNECs was significantly downregulated, indicating that PNECs were likely the primary source of CGRP. Administration of rimegepant, a CGRP receptor antagonist, led to reductions in inflammatory cells, decreased mucus-producing cell hyperplasia, and lowered both ILC2 counts and IL-5 production [124].

These findings suggest that PNECs may play a role in asthma by enhancing allergen-induced type 2 immune responses. Additionally, targeting ILC2 with an anti-CGRP signal strategy could be considered a potential therapy for allergic asthma [123,124].

### 5.6. Tuft Cells in Healh and in Asthma

TFcs, also known as solitary chemosensory cells (SCCs) in the sinonasal area [125,126], brush cells in the trachea [127], microvillous cells in the human olfactory epithelium [128], and TFcs in the intestinal tract, are characterized by a distinct microvillous apical tuft [55].

TFcs are mainly studied in the gut, where their IL-25 supports ILC2 cells. ILC2s then produce IL-13, which drives TFc differentiation and proliferation via IL-4Rα. This ILC2-TFc circuit recruits eosinophils, leads to GC hyperplasia, and aids helminth expulsion, indicating TFcs’ role in type 2 inflammation through a TFc-ILC2-IL-13 pathway [129,130,131].

Researchers have identified immature tuft, tuft-1, and tuft-2 cells among murine tracheal TFcs. Tuft-1 cells are linked to taste signaling, while tuft-2 cells relate to leukotriene synthesis and immune modulation, indicating chemosensory and immunological roles, respectively [55]. Airway TFcs produce IL-25 and TSLP [55], with tuft-2 cells particularly expressing genes for leukotriene biosynthesis like *Alox5ap* [132].

TFcs in the human olfactory epithelium and airways express both sweet/umami (T1R) and bitter (T2R) taste transduction components [55].

TFcs boost antimicrobial responses by activating nearby epithelial cells through bitter taste receptors, which detect bacterial products and support innate immunity [133,134]. When TFcs in the upper airway are exposed to T2R receptor agonists like denatonium benzoate, parthenolide, or amarogentin, an intracellular calcium wave triggers the release of antimicrobial substances [135,136] and cyteinyl leukotrienes [133,137].

Choline acetyltransferase (ChAT) is present in most TFcs, including those in the human respiratory tract [138]. Signals like bitter compounds, cigarette smoke, and bacterial metabolites trigger TFcs to produce acetylcholine [138,139]. Acetylcholine supports epithelial homeostasis, MCC, and smooth airway muscle regulation in both upper and lower airways [140,141].

Analysis of scRNA-seq data from human sinonasal cells showed that TFc markers are elevated in the nasal cavity of CRS patients [142]. TFcs in the sinonasal epithelium produce IL-25 after IL-13 treatment [142]. Additionally, IL-25 exposure activates type 2 innate lymphoid cells and Th2 cells in nasal polyp cells, boosting Th2 cytokine production in vitro. These findings suggest TFcs may play a role in CRSwNP pathogenesis [143].

IL-25 plays a crucial role in virus-induced asthma exacerbations [144]. In patients with allergic asthma, plasma IL-25 levels are elevated [145]. Moreover, sputum IL-25 concentrations are higher in atopic than non-atopic asthma and correlate with disease severity [146]. These findings suggest that TFcs, which are associated with CRS, a common comorbidity of severe asthma, may also be involved in asthma. However, there is limited data on TFcs in the airways of patients with asthma.

A recent study of 154,222 AECs from non-asthmatic donors found ionocytes in 0.45% and mature TFcs in only 0.002% [147]. Researchers identified a new bipotent progenitor cell, termed tuft-ionocyte progenitor (TIP) cells, which can differentiate into both ionocytes and TFcs. TIP cells primarily develop into ionocytes, which explains the scarcity of mature TFcs in airways. However, Type 2 and Type 17 cytokines, associated with asthma, shift TIP cells towards TFc differentiation in vitro. Consistent with this finding, mature TFcs were identified in the airways of a patient who died from an asthma exacerbation. This suggests that immune signaling in asthma may alter the rare cell composition related to the disease [147]. Further research is needed to understand the role of TFcs in affecting IL-25 and ILC2s in the airways of patients with asthma

### 5.7. Pulmonary Ionocytes in Health and in Asthma

In 2018, scRNA-seq identified a novel cell type in the epithelial lining of the respiratory tract, known as ionocytes [55,144]. PIs, present in both mice and human airways, are derived from BC precursors or tuft-like cells [55,144].

Ionocytes express elevated levels of transcripts encoding the cystic fibrosis transmembrane conductance regulator (CFTR) protein, an anion channel defective in patients with cystic fibrosis (CF) [145]. CFTR is localized to the apical surface of airway epithelial cells (AECs) and is responsible for the transport of chloride and bicarbonate ions, whose concentrations determine the tonicity and pH of the airway fluid, both of which are essential parameters for effective mucociliary transport and host defense [145]. Early studies found that PIs account for roughly 55% of CFTR transcripts, while more abundant CLCs, BCs, and CCs also express low levels of CFTR, suggesting that the receptor may have a cell-type-specific role. Ionocytes make up less than 2% of human AECs, express FOXI1, and depend on NOTCH signaling for differentiation [55,144].

However, recent studies using scRNA-seq technologies, coupled with more sensitive molecular methods, have identified secretory cells dominating CFTR expression in normal human large and small airway superficial epithelia, followed by BCs [146]. Ionocytes expressed the highest levels of CFTR per cell, which is consistent with earlier reports [55,144], while expression in CLCs was low. CFTR-mediated chloride secretory function was correlated with secretory cell types but not ionocytes. This suggests that secretory cells primarily mediate CFTR expression, thereby contributing to the well-hydrated mucus on airway surfaces [146].

These findings were not replicated in another study examining the ion transport properties of PIs and CCs at the single-cell level in primary human bronchial epithelial cell cultures from donors with and without CF [147]. The study found that activation of the Sonic Hedgehog (SHH) signaling pathway is increased in CF. SHH signaling is critical in regulating cell proliferation and differentiation [148]. Inhibiting the SHH pathway affects airway cell differentiation in a cell-type-specific manner [148]. The study reported increased SHH activity resulting in a higher number of mature ionocytes (BSND-barttin-positive ionocytes), which correlated with enhanced CFTR activity. Pharmacological inhibition of SHH resulted in reduced ionocyte numbers, CFTR protein expression, and CFTR activity. Conversely, SHH inhibition led to increased secretory cell numbers but less CFTR activity, proving a discordant relationship between increased secretory cell numbers and the resulting CFTR activity. Differences in the epithelial cell samples and methods used may account for the dissimilarities seen between this and previous studies [147].

A subsequent study revealed that ionocytes in various genetic ferret models control airway surface liquid (ASL) absorption, secretion, pH, and mucus viscosity, affecting CF. These processes are regulated by CFTR-dependent ionocyte transport of Cl− and HCO3−. Three subtypes of PIs (A, B, and C) and a FOXI1-lineage common progenitor for ionocytes, TFcs, and PNCs were identified. Type C ionocytes appear to be the progenitors of type A and B ionocytes. These findings suggest that rare PIs play essential CFTR-dependent roles in the proximal airway characteristics of CF airway disease [148]. Hillock basal stem cells prove the capacity for significant clonal expansion, which is adequate to resurface denuded airways and ultimately regenerate normal airway epithelium, including all six of its component cell types [149].

Unraveling the functional role of ionocytes in asthma has been the aim of some recent studies [150,151].

Human BECs were obtained from endobronchial brushes of airways from patients with non-eosinophilic asthma. The epithelia showed a decrease in ASCL3 and FOXI1 double-positive cells, a population that includes both mature and immature ionocytes. There was a decrease in the number of mature FOXI1+ ionocytes that expressed CFTR [151].

The authors also explored the relationship between ionocyte numbers and inflammation by treating epithelial cells derived from healthy human airways with Type 1 and Type 17 cytokines. A combination of TNF-α, interferon gamma, IL-17A, and IL-22 resulted in a significant reduction in both total (FOXI1+ ASCL3+) and mature (FOXI1+ CFTR+) ionocytes. Treatment with individual cytokines IL-17A and interferon gamma also led to considerable reductions in mature CFTR-expressing ionocytes. The study showed that CFTR modulator therapy does not improve CFTR function in non-eosinophilic epithelia, due to the presence of fewer ionocytes. The lack of ionocytes may reduce airway surface fluid in patients with asthma, leading to mucus plugs and airway obstruction [150].

Another recent study demonstrated that human bronchial airway epithelia treated with IL-13 displayed fewer ionocytes with reduced CFTR expression, suggesting that the cytokine may affect the number of ionocytes and their CFTR regulatory function. Additionally, IL-13-stimulated epithelia displayed an overall increase in CFTR expression due to an increase in CFTR-expressing GCs and a concordant increase in CFTR-mediated chloride function, despite the loss of ionocytes. Therefore, IL-13 has opposing effects on CFTR regulation in a cell-type–specific manner. IL-13 increases the contribution from secretory cells to epithelial CFTR function while decreasing the contribution from ionocytes to epithelial CFTR function. These changes were associated with an increase in ASL volume. Reduced CFTR expression in ionocytes and increased CFTR-expressing GCs are linked to severe airflow obstruction, suggesting that Th2 inflammation might result in the production of more mucus, which is further exacerbated by the increased airway hydration associated with reduced fluid absorption, resulting in mucous hypersecretions contributing to airway obstruction [151].

Collectively, these two studies indicate that the loss of CFTR in ionocytes occurs in both non-eosinophilic and eosinophilic asthma, potentially contributing to their pathophysiology.

### 5.8. Hillock Cells in Health and in Asthma

Hillock cells are characterized as a population of basal progenitor cells expressing keratin 13 (KRT13) and TP63 [55,149]. These cells are found in contiguous groups of stratified epithelial cells, forming structures termed ‘hillocks’, where they have a particularly high turnover rate and express markers associated with squamous epithelial differentiation, cellular adhesion, and immunomodulation, including Scgb1ab1+ and KRT13+ [55,149]. Hillock basal stem cells are capable of massive clonal expansion, which is sufficient to resurface the denuded airway and eventually regenerate normal airway epithelium with each of its six component cell types [152].

Hillock cells are known to regenerate normal airway epithelium [152]; however, their potential role in repairing the altered epithelium in patients with asthma is unknown.

### 5.9. Airway Cell Adhesions in Health and in Asthma

Cell–cell adhesions are crucial for tissue development and maintenance. Airway cells coordinate movements during development and wound repair, requiring cytoskeleton and cell junction remodeling driven by intercellular signaling [153].

Airway cells are connected by various junctional complexes, such as tight junctions (TJs), adherens junctions (AJs), gap junctions (GJs), desmosomes, hemidesmosomes, and focal adhesions. These junctions are organized with the TJ closest to the apical (lumen-exposed) part of the cell, followed by the AJ just below it. GJ, desmosomes, hemidesmosomes, and focal adhesions are found further toward the basal side [153].

Cellular junctions can be functionally grouped into occluding (i.e., TJs), anchoring (i.e., AJs, desmosomes, and hemidesmosomes), and communicating junctions (i.e., gap junctions) [153].

#### 5.9.1. Tight Junctions

TJ proteins include claudins, occludins, the Zona occludens (ZO-1, ZO-2, and ZO-3), TJ-associated MARVEL (MAL and related proteins for vesicle trafficking and membrane link) proteins (TAMPs), and junctional adhesion molecules (JAMs). These proteins maintain barrier function by binding to actin fibers and each other in the cytoplasm [154]. Due to their apical position, TJs manage the paracellular passage of ions and macromolecules across the epithelium. Water and ion permeability are mainly controlled by the claudin protein family, while macromolecules are primarily regulated by the TAMP family [155,156]. The TAMP family contains occludin, tricellulin, and marvelD3, all characterized by the four-pass transmembrane MARVEL domain [156].

Claudins are tetraspan transmembrane proteins that play a crucial role in tight junction permeability. They consist of four transmembrane domains, N- and C-terminal domains, two extracellular domains, and a short intracellular loop. In humans, there are 27 types of claudins, categorized into two groups: classic and non-classic. Claudins interact within the same membrane (homomeric cis-interactions) and between cells (homotypic trans-interactions) to form functional barriers. Heteromeric or heterotypic interactions occur between different claudins within the same cell or between adjacent cells. The roles of these heteromeric and/or heterotypic interactions in supporting a healthy barrier in the human lung are yet to be found [157,158].

Claudins are further classified depending on whether they create paracellular channels (pore-forming) or limit permeability (sealing claudins). Sealing claudins play a crucial role in preventing the uncontrolled leakage of water, ions, and metabolites between the apical and basolateral compartments of the lung epithelium [157,158].

Occludin, unlike claudins, is not essential for TJ assembly but is crucial for maintaining their stability and barrier function [159]. Occludin spans the membrane four times, forming two extracellular loops and cytoplasmic N- and C-termini. The first loop facilitates interactions between cells, while the second ensures the localization of occludin to TJs, aiding in its interaction with claudins [160,161]. The C-terminus also binds various cytoplasmic proteins, including ZO proteins. Decreased lung occludin has been associated with various conditions known to promote pulmonary edema [162,163].

ZO proteins, including ZO-1, -2, and -3, are proteins that localize at junctional sites. They act as scaffolding proteins, recruiting various proteins to the cytoplasmic surface of the junction, forming the “junctional plaque”. Initially described as TJ-specific (*zonulae occludentes*), ZO proteins interact with transmembrane proteins like occludin, claudins, JAM, tricellulin, and CAR (Coxsackievirus and Adenovirus Receptor), indicating their key role in intercellular adhesion and communication [164,165].

Studies indicate that IL-4 and IL-13, key cytokines in asthma, disrupt TJ components, impairing barrier function in asthma [166,167]. An in vivo mouse model revealed that IL-13 from ILC2s leads to bronchial epithelial barrier disruption by regulating TJs in experimental asthma pathogenesis [168]. Specifically, IL-13 reduces claudin-18 production, a crucial TJ component, decreasing transepithelial electrical resistance and increasing epithelial permeability in asthma [169].

Occludin and ZO-1 are also downregulated in mouse models of eosinophilic asthma [170]. Likewise, BECs from patients with asthma exhibit decreased expression of these proteins compared with those from healthy subjects [147].

A recent study revealed that β-eudesmol (BE), a bioactive compound isolated from the rhizome of *Atractylodes lancea*, exhibits an anti-allergic inflammatory effect on the TJ of the airway epithelium. This effect is linked to the inhibition of IL-4/IL-13-induced TJ barrier disruption. BE prevented the IL-4/IL-13-induced mislocalization of TJ components, including occludin and ZO-1. Furthermore, research showed that BE’s ability to protect tight junctions is linked to its inhibition of STAT6 phosphorylation triggered by IL-4 and IL-13 [171].

Long-term fluticasone furoate (FF) increased the expression of occludin in patients with AR compared with controls [157]. In an HDM-induced allergic asthma mouse model, FF and mometasone furoate had positive effects on mucosal permeability and increased the mRNA expression of occludin and ZO-1 [157]. These findings indicate that the efficacy of corticosteroids in rhinitis and asthma may be partly due to their ability to restore the normal function of TJs.

Patients with asthma had higher plasma junction adhesion molecule-A (JAM-A) levels compared to the control group. Additionally, JAM-A levels showed correlations with clinical variables, including lung function, total IgE, and blood lymphocyte proportions, among patients with asthma [172].

#### 5.9.2. Adherens Junctions

AJs feature two main types of transmembrane receptors: cadherins and nectins. The extracellular domains of cadherins enable cell adhesion, while the intracellular domains interact with proteins that regulate connections to the actin cytoskeleton and activate signaling pathways [173].

The cadherin superfamily consists of more than 20 members and includes epithelial-cadherin (E-cad), N-cadherin, P-cadherin, desmosomal cadherin, and protocadherin (PCDH) [173,174]. E-cad has five immunoglobulin-like extracellular repeated domains that help cell adhesion, thereby preventing cell separation [130]. The cytoplasmic tail of E-cadherin mediates interactions with catenin proteins and other actin cytoskeletal binding proteins. The interaction between E-cadherin and α, β, and p120 catenins stimulates the Rho family of GTPases, which later modulate actin dynamics [173].

The nectins, a family of four immunoglobulin superfamily members (nectin-1 to -4), interact through their extracellular domains to support cell–cell adhesion and help establish apical-basolateral polarity. The extracellular region comprises three Ig-like loops, a single transmembrane region, and a cytoplasmic tail region [174]. In epithelial cells, they localize exclusively at AJs, which mechanically connect neighboring cells to resist strong contractile forces. Nectins interact with cadherins through their binding proteins, afadin and β-catenin/αE-catenin, linking them to the actin cytoskeleton. However, nectin dimers do not support strong adhesion, making cadherins the primary receptor in AJs. Nectins can engage in trans-interactions with the same or different nectin family members, forming homophilic and heterophilic trans-interactions through their extracellular regions [174].

Loss of cell adhesion molecules, like E-cadherin, may be key in asthma development [175]. Studies show that silencing E-cadherin with siRNA reduces epithelial resistance and increases the expression of TSLP and C-C motif chemokine ligand 17 [176].

PCDH1 (Protocadherin 1) colocalizes with E-cadherin at the apical site of AECs, and its expression increases during the differentiation of cultured AECs [129,130]. Immunohistochemical analysis identified strong PCDH1 expression in nasal and bronchial epithelial cells, though the expression decreased in inflamed tissues from patients with CRS or bronchial asthma. Dexamethasone enhanced the barrier function of AECs and increased PCDH1 expression. These findings suggest that PCDH1 dysfunction may be involved in the pathogenesis of airway inflammation, and that glucocorticoids may promote epithelial barrier integrity by inducing PCDH1 expression [177].

Plasma nectin-4 levels were higher in patients with asthma than in controls and correlated with lung function. Nectin-4 increased in normal human BECs exposed to the *Dermatophagoides pteronyssinus* 1 allergen but decreased with Nectin-4 siRNA treatment. In mice sensitized/challenged with OVA, airway obstruction and inflammation, along with Nectin-4 and Th2 cytokine levels, were higher in wild-type than in sham mice. Nectin-4 knockdown resulted in lower levels of airway inflammation and dysfunction in OVA-induced asthma [178].

Enfortumab vedotin—an antibody-drug conjugate targeting nectin-4— is used in the treatment of patients with urothelial carcinoma. Asthma exacerbation following enfortumab vedotin treatment has been recently documented [179].

Collectively, these findings indicate that nectin-4 may play a role in airway inflammation and dysfunction in asthma

#### 5.9.3. GAP Junctions

Gap junctions help direct interaction between adjacent cells through intercellular channels. In humans, these channels consist of proteins encoded by the 21-member connexin gene family. Connexin proteins form channels that allow the transfer of small molecules, such as ions, metabolites, and secondary messengers, which are essential for keeping cellular homeostasis [180].

Six connexins form a connexon, which joins another connexon on a neighboring cell to create a gap junction channel. Connexons can also function as hemichannels. Initially thought to be intermediate in forming gap junctions, hemichannels have been found to exist independently and participate in autocrine and paracrine signaling. Connexins have short half-lives (1–4 h) and are regulated to rapidly respond to environmental changes or cellular growth [181,182,183].

Gap junctions play a role in innate immunity by transmitting pro-inflammatory and proapoptotic signals between cells through activated pathogen recognition receptors. During inflammation, connexin hemichannels and gap junctions are typically regulated in different ways: hemichannels tend to open, whereas gap junctions often decrease [184].

Over twenty different types of connexins have been identified in humans, such as Cx26, Cx32, Cx37, Cx40, Cx43, and Cx46 [182,183,184,185]. Cx43 is among the most broadly distributed gap junction proteins and can be found in many cell types, such as epithelial cells [183]. Cx43 can interact with proteins involved in other pathways, thereby affecting processes such as cellular proliferation, apoptosis, and inflammation [183].

Pannexins are transmembrane proteins identified in the year 2000 and include three types: Panx1, Panx2, and Panx3. Despite differing from connexins in amino acid sequences, their membrane structure is similar, forming hexameric hemichannels. Pannexin 1 is present in all tissues, pannexin 2 is mainly in the central nervous system, and pannexin 3 is in bone, cartilage, and skin. These hemichannels connect the cytoplasm to the external environment, releasing low-molecular-weight molecules such as purines for paracrine signaling [186].

Panx1 hemichannels activate inflammasomes in non-immune cells like AECs [187], and facilitate communication between epithelial cells and macrophages, contributing to epithelial regeneration after injury [188].

Emerging evidence suggests that connexins and pannexins have biological functions beyond channel activity [189,190,191,192,193,194]. Research on gap junctions in asthma has mainly focused on the role of Cx43 in allergic asthma [189,190,193]. Cx43 is upregulated in an OVA-induced allergic lung inflammation model, with its levels correlating with increased inflammation. The inhibition of Cx43 reduced key features of allergic asthma, such as eosinophil infiltration, Th2 cytokine levels, and airway hyperresponsiveness [189]. However, the affected cell type remains unclear. Cx43 is expressed by airway epithelium and inflammatory recruited cells, such as mast cells [190]. Thus, it is uncertain whether the diminished allergic response results from impaired Cx43 expression in AECs, inflammatory cells, or both [191].

Pannexins might help reduce inflammation severity. Excessive ATP release into ASL from patients with asthma signals inflammation and blocking Panx1 channels prevented airway hyperreactivity in an asthmatic mouse model [193].

A recent study showed that Panx1-mediated intercellular communication between T-regulatory and T-effector lymphocytes break down ATP to adenosine via ectonucleotidases, with adenosine showing an anti-inflammatory effect in mice challenged intranasally with dust mite allergens [194].

#### 5.9.4. Desmosomes, Hemidesmosomes, and Focal Adhesions

Desmosomes are cellular structures in tissues, such as cardiac muscles and epithelia, which endure mechanical stress. These junctions keep cells together, affording mechanical strength and structural stability [195,196].

Desmosomes are composed of a protein complex that includes multiple proteins organized into three main regions: (a) Desmosomal plaque: A dense network of proteins located on the cytoplasmic side of the membrane, connecting cytoskeletal intermediate filaments to transmembrane proteins. (b) Transmembrane proteins: Including desmosomal cadherins, such as desmoglein and desmocollin, which enable adhesion between adjacent cells. (c) Intermediate filaments: These anchor the desmosomal plaque, providing resistance to mechanical stress [195,196].

Desmosomes serve three main functions: (a) Cell cohesion, which maintains tissue integrity, as in epithelial cells; (b) Transmission of mechanical forces, distributing stress to prevent cell rupture; and (c) Cell signaling, involved in regulating processes such as cell proliferation and differentiation [195,196]. A banded desmosome is a desmosome arrangement in certain epithelial cells that creates an adhesive belt around the cell [195,196].

Hemidesmosomes create strong links between cells and the ECM, crucial for epithelial tissues. They connect epithelial cells to the basal lamina using integrins and plectin that bind to intermediate filaments, thereby enhancing tissue stability and affecting various cellular processes [197,198].

Focal adhesions connect integrins and actin filaments, helping cells adhere to the ECM. Integrins, made of one beta and one alpha subunit, are classified into four groups based on their ligands: RGD, laminin, leukocyte-specific, and collagen receptors. Inside the cell, the intracellular domain of integrin links to the cytoskeleton through adapter proteins, like talin, α-actinin, filamin, vinculin, and tensin. Other signaling proteins, including focal adhesion kinase, can initiate a phosphorylation cascade activating various downstream targets [199].

Very little is known about the role of these anchoring junctions in asthma. Transmission electron microscopy studies revealed that the relative length of desmosome connections between columnar and basal cells was shorter in patients with both allergic and non-allergic asthma compared with healthy controls, implying that patients with asthma may have desmosomal weakening [200,201,202,203,204].

## 6. Conclusions

Airway epithelial cell dysfunction has been widely acknowledged as a major contributor to the development of asthma [2,53,54,202,203,204,205,206,207,208,209]. Despite this assumption, most research over the last two decades has focused on studying the complex and extended inflammatory response resulting from the disturbed epithelium.

Research using omics has revealed the complex molecular processes behind asthma [10,11,12]. Our review of the most representative studies conducted with omics shows the reported activation of hundreds of genes, with only a few replicated in more than one study. It remains uncertain whether the majority of DEGs that are regulated differently across various asthma endo/subendotypes play significant roles or are merely incidental.

If asthma initiates in the altered epithelium, focusing research on epithelial dysfunction is logical. Variations in the characteristics of the disturbed airway epithelium, resulting in different responses to external signals, may contribute to the diversity of asthma endotypes.

Studies have proved the complex cellular structure of the airway epithelium, revealing new cells with significant regulatory functions [55,56,57,144]. Cellular junctions link airway cells together, which is essential for maintaining lung homeostasis [199]. Various factors can disrupt this structure, triggering inflammation that worsens airway damage and leads to distinct asthma phenotypes and endotypes. Knowledge about the role of airway cells and cellular junctions in the varied clinical and inflammatory presentation of asthma is still in its infancy.

Recent advances in scRNA-seq enable researchers to analyze the RNA expression profiles of individual cells. This technique helps identify and characterize cell types, understand cell-to-cell variation, and study gene regulation in the airways of asthma patients [55,56,57,144]. Additionally, the use of biologics targeting airway epithelial cytokines, such as alarmins, will provide relevant information to identify whether alterations are the cause or consequence of disturbed airways [206].

The latest goal in asthma therapy is to achieve remission with potent biologics [210]. Asthma remission is mainly determined using clinical criteria and assessments of lung function [211,212]. Nevertheless, focusing on repairing the damaged airways may lead to a more ambitious aim: maximal restoration of the damaged airway. The best therapy will be that which restores the epithelium to its normal state, or as close to normal as possible.

In summary, growing consensus focuses asthma research on the airway epithelium, recognizing its key role in diagnosis, management, and disease progression. Understanding how epithelial dysfunction contributes to different asthma types is essential for developing more effective treatments and improving patient outcomes [53].

## Data Availability

No new data was created or analyzed in this study.

## Abbreviations

The following abbreviations are used in this manuscript:

Adherens junctions	AJs
Airway epithelial cells	AECs
Alkaline phosphatase tissue-nonspecific isozyme	ALPL
Basal cell	BC
Body mass indexBMI	BMI
Bronchial epithelial cells	BEC
Bronchoalveolar lavage fluid	BALF
Calcitonin gene-related peptide	CGRP)
Carboxypeptidasa A3	CPA3
Chloride chanel regulator 1	CLCA1
Charcot-Leyden crystal protein	CLC
Chemokine C-X-C motif receptor 2	CXCR2
Ciliated cell	CLC
Clara cell 16	CC16
Club cell	CC
Chronic obstructive pulmonary disease	COPD
Chronic rhinosinusitis with nasal polyps	CRSwNP
Cystic fibrosis transmembrane conductance regulator	CFTR
Dendritic cells	DCs
Differentially expressed genes	DEGs
Extracellular matrix	ECM
Forced expiratory volume 1 s	FEV1
Fractional exhaled nitric oxide	FeNO
Gap junctions	GJs
Goblet cell	GC
Inhaled corticosteroid	ICS
Innate lymphoid cells	ILCs
Interferon gamma	IFN-γ
Interleukin	IL
Junction adhesion molecule-A	JAM-A
Mucociliary clearance	MCC
Nucleotide-binding domain and leucine-rich repeats containing pyrin domain 3	NLRP3
Omics-associated clusters	OACs
Periostin	POSTN
Pregnancy-associated plasma protein	A PAPP-A
Prostaglandin	PG
Protocadherin 1	PCDH1
Pulmonary ionocyte	PI
Pulmonary neuroendocrine cell	PNEC
Reactive oxygen species	ROS
Serpin peptidase inhibitor clade B member 2	SERPINB2
Single-cell RNA sequencing	ScRNA-sep
Thymic stromal lymphopoietin	TSLP
Tight junctions	TJs
Toll-like receptor	TLR
Tuft cell	TC
Tumor necrosis alfa	TNF-α
Zona occludens	ZO
